# Inhibition of Aspirin-Induced Gastrointestinal Injury: Systematic Review and Network Meta-Analysis

**DOI:** 10.3389/fphar.2021.730681

**Published:** 2021-08-12

**Authors:** Wan-tong Zhang, Miao-ran Wang, Guo-dong Hua, Qiu-yan Li, Xu-jie Wang, Rui Lang, Wei-liang Weng, Chun-miao Xue, Bao-chen Zhu

**Affiliations:** ^1^ Institute of Clinical Pharmacology, Xiyuan Hospital, China Academy of Chinese Medical Sciences, Beijing, China; ^2^ Department of Endocrinology, Xiyuan Hospital, China Academy of Chinese Medical Sciences, Beijing, China; ^3^ Department of Pharmacy, Dongzhimen Hospital, Beijing University of Chinese Medicine, Beijing, China; ^4^ National Clinical Research Center for Chinese Medicine Cardiology, Beijing, China; ^5^ Department of Nephrology, Xiyuan Hospital, China Academy of Chinese Medical Sciences, Beijing, China

**Keywords:** aspirin, gastrointestinal injury, healthy subject, network meta-analysis, randomized controlled trials

## Abstract

**Background:** Administration of aspirin has the potential for significant side effects of gastrointestinal (GI) injury mainly caused by gastric acid stimulation, especially in long-term users or users with original gastrointestinal diseases. The debate on the optimal treatment of aspirin-induced gastrointestinal injury is ongoing. We aimed to compare and rank the different treatments for aspirin-induced gastrointestinal injury based on current evidence.

**Methods:** We searched PubMed, EMBASE, Cochrane Library (Cochrane Central Register of Controlled Trials), and Chinese databases for published randomized controlled trials (RCTs) of different treatments for aspirin-induced gastrointestinal injury from inception to 1 May 2021. All of the direct and indirect evidence included was rated by network meta-analysis under a Bayesian framework.

**Results:** A total of 10 RCTs, which comprised 503 participants, were included in the analysis. The overall quality of evidence was rated as moderate to high. Eleven different treatments, including omeprazole, lansoprazole, rabeprazole, famotidine, geranylgeranylacetone, misoprostol, ranitidine bismuth citrate, chili, phosphatidylcholine complex, omeprazole plus rebamipide, and placebo, were evaluated in terms of preventing gastrointestinal injury. It was suggested that omeprazole plus rebamipide outperformed other treatments, whereas geranylgeranylacetone and placebo were among the least treatments.

**Conclusion:** This is the first systematic review and network meta-analysis of different treatments for aspirin-induced gastrointestinal injury. Our study suggested that omeprazole plus rebamipide might be considered the best option to treat aspirin-induced gastrointestinal injury. More multicenter, high quality, large sample size randomized controlled trials will confirm the advantages of these medicines in the treatment of aspirin-induced gastrointestinal injury in the future.

## Introduction

Aspirin is a well-known baseline antiplatelet agent, serving as the primary and secondary prevention as well as the treatment of ischemic stroke, acute myocardic infarction, transient ischemic attack, acute coronary syndrome, and peripheral artery diseases (A. J. Hermosillo and Spinler, 2008). However, many studies have shown that the administration of aspirin has the potential for significant side effects of gastrointestinal (GI) symptoms including mucosal lesions, bleeding or peptic ulcers, especially in long-term users or users with origin gastrointestinal diseases (R. Guthrie, 2011). The aspirin-induced gastric injury is a chronic inflammation mainly caused by gastric acid stimulation, as aspirin break the gastric mucosal barrier; gastric acid is capable of breaking the mucosal epithelial cells directly, causing inflammation, bleeding, and gastric ulcer. However, the mechanism of aspirin-induced small bowel injury has remained incompletely understood; its occurrence involves intestinal microorganism, bile and other stimulating factors, which is more complex than the gastric injury caused by aspirin (Washio, et al., 2016; Wallace, 2012). Therefore, there are no guideline recommendations for the treatment of aspirin-induced gastrointestinal injury.

Medications such as proton pump inhibitors (PPIs), histamine 2 receptor antagonists (H2RAs), misoprostol, and alendronate are used clinically to prevent aspirin-induced gastrointestinal injury. Recent randomized controlled trials (RCTs) have demonstrated that these treatments have high therapeutic potential effects for aspirin-induced gastrointestinal injury. However, it is difficult to directly rank the efficacy and safety of the treatments due to the limitations of study design and scale.

Comparing to conventional meta-analysis, network meta-analysis (NMA) offers more advantages, which has been increasingly advocated in medical research, as it provides more comprehensive analytical evidence for selecting the optimal treatment by directly and indirectly evaluating different intervention models (Nikolakopoulou et al., 2018). To address this knowledge gap, we conducted a systematic review and network meta-analysis of randomized controlled trials (RCTs) of all the medications used in aspirin-induced gastrointestinal injury to evaluate their relative efficacy and safety.

## Methods

Our systematic review and NMA manuscript was written in accordance with the Cochrane Handbook (Higgins et al., 2020). Reporting was consistent with the Preferred Reporting Items for Systematic Reviews and Meta-Analysis (PRISMA) extension statement for reporting systematic reviews incorporating NMA (Guise et al., 2017).

### Data Sources and Searches

We searched PubMed, EMBASE, Cochrane Library (Cochrane Central Register of Controlled Trials), and two Chinese databases (China National Knowledge Internet database and China Wanfang database) for citations published in any language from inception to 1 May 2021. We used combinations of MeSH terms and text words around “aspirin,” “acetylsalicylic acid,” “gastrointestinal injury,” “gastrointestinal lesions,” “peptic ulcer,” “bleeding,” “perforation,” “obstruction,” “healthy people,” “healthy subjects,” “randomized controlled trial,” and “clinical trial.” Additional studies were derived from screening the reference lists of included RCTs and previous systematic reviews. We also searched trial registers like ClinicalTrials.gov, iSCTRN, and OpenTrials.net to identify any unpublished or ongoing trials and contacted the researchers to ask about unpublished studies.

### Eligibility Criteria

We included the RCTs with the following criteria: *1*) RCTs inhibiting aspirin-induced gastrointestinal injury with medication in healthy people; *2*) RCTs reporting number of people with gastrointestinal lesions, with or without safety outcome; *3*) minimum sample size no less than 20.

We excluded the RCTs with the following criteria: *1*) RCTs with significant flaws, incomplete or wrong results; *2*) dissertations and conference abstracts; and *3*) RCTs with the participant numbers in the smaller group less than 20. Besides, we excluded observational, cross-sectional, case series, or qualitative studies.

### Data Extraction and Quality Assessment

Two independent authors (Wan-tong Zhang and Bao-chen Zhu) assessed all RCTs for eligibility and extracted data and assessed study quality. Disagreements were resolved by consensus-based discussion. The full-text articles were downloaded and the same inclusion criteria were used to decide whether to include or exclude at this stage. The average age and sex ratio of patients, grouping, intervention measures and outcome indicators were recorded. Each reviewer independently carried out the quality assessment of each selected article, assessed the completeness of the data extraction, and confirmed the quality rating to reduce bias.

Three authors (Wan-tong Zhang, Bao-chen Zhu, and Miao-ran Wang) evaluated all the included RCTs. Any disagreement was solved through discussion, mutual consensus, and rechecking of the article. For the RCTs which were included, the Cochrane risk-of-bias tool was used to assess risk of bias (Sterne et al., 2019), including selection bias (random sequence generation/allocation concealment), performance bias (blinding of participants and personnel), detection bias (blinding of outcome assessment), attribution bias (incomplete outcome data), reporting bias (selective outcome reporting), and other possible sources of bias. In this way, all articles selected for inclusion in the review were graded under the categories of low, high, or unclear risk of bias.

The efficacy outcome was the number of individuals diagnosed with gastrointestinal injury after taking aspirin. Safety assessments included adverse events (AEs).

### Data Synthesis

For further evaluation of gastrointestinal protecting medications simultaneously, a network meta-analysis was used to synthesize the study effect sizes (both direct and indirect comparisons). Heterogeneity across individual studies was estimated by the Cochran Q test (Chi-squared) and Higgins I-squared inconsistency statistic. If there was no significant heterogeneity (*p* > 0.05, I-squared <50%), a fixed-effects model was used. Otherwise, a random-effects model was used. Moreover, the analysis of ranking probabilities and the Surface under the Cumulative Ranking curve (SUCRA) were used to explore the probabilities of each treatment being ranked the best. The SUCRA value, expressed as a percentage, is one of the best choices for indicating the relative probability of intervention. Inconsistency in the network was evaluated using the deviance information criterion (DIC) between the consistent and inconsistent models. If the DIC difference was within 3, the data were generally considered to be consistent. Once considerable heterogeneity (*p* < 0.05, I-squared>50%) was found in pairwise meta-analyses, we will conduct a sensitivity analysis; that is, we will remove those studies that lead to high heterogeneity indirect comparisons or introduced statistical inconsistency in the NMA. All data were analyzed by using the R 3.6.3 software and Review Manager version 5.4.

## Results

### Study Screening

**FIGURE 1:**
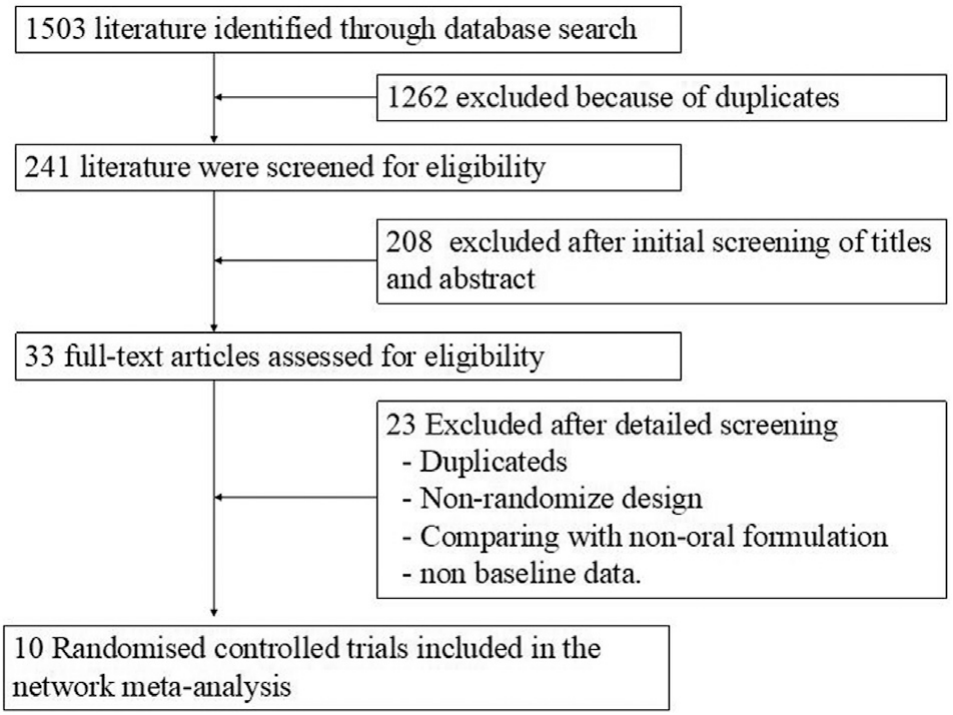
Flow chart indicating the selection process for this network meta-analysis.

By searching five different electronic databases and manual reference searching, 241 relevant publications were found after removing duplications and unrelated studies. Relevant articles were further selected by reading the full texts. By excluding publications that contained noneligible data, had unsuitable comparator arms, and were not clinical trials and nonrandomized trials, 10 articles were included. Figure 1 shows the detailed steps of the literature selection process.

### Study Characteristics and Network Plots

**FIGURE 2:**
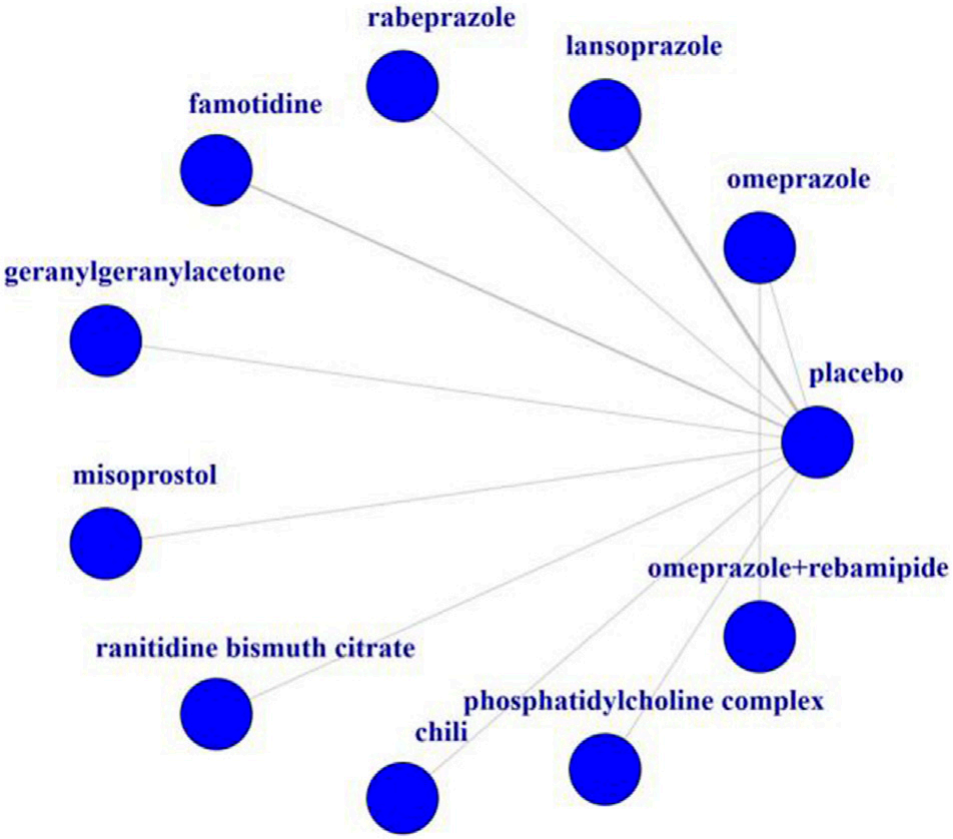
Network of eligible comparisons for the meta-analysis.

In total, 10 trials were included in the NMA, and their basic characteristics are presented in Table 1. A network of eligible comparisons for the multiple-treatment meta-analysis was constructed (Figure 2). These RCTs compared eleven treatments, namely omeprazole, lansoprazole, rabeprazole, famotidine, geranylgeranylacetone, misoprostol, ranitidine bismuth citrate, chili, phosphatidylcholine complex, omeprazole plus rebamipide, and placebo.

**TABLE 1:** Characteristics of RCTs included in the analysis.

Study	Country	Intervention group		Control group		Treatment duration (days)	Outcomes	Side effect	Conclusion
		Sample	Treatment	Sample	Treatment				
Kazuhiro et al. (2011)	Japan	11	Aspirin (100 mg/day) + omeprazole (20 mg/day) + rebamipide (300 mg/day)	11	Aspirin (100 mg/day) + omeprazole (20 mg/day)	28	1. Number of participants with gastrointestinal lesions	None	The combination use of rebamipide and omeprazole is superior to omeprazole
							2. Side effect		
Sugimoto et al. (2012)	Japan	15	①Aspirin (100 mg/day) + famotidine (40 mg/day)	15	Aspirin (100 mg/day)	7	1. Number of participants with gastrointestinal lesions	None	Lansoprazole and rabeprazole are superior to famotidine
			②Aspirin (100 mg/day) + lansoprazole (15 mg/day)						
			③Aspirin (100 mg/day) + rabeprazole (10 mg/day)				2. Side effect		
Byron et al. (2011)	USA	90	Aspirin–phosphatidylcholine complex (325 mg/day)	91	Aspirin (325 mg/day)	7	1. Number of participants with gastrointestinal lesions	None	Aspirin–phosphatidylcholine complex is superior to placebo
							2. Side effect		
Masafumi et al. (2011)	Japan	15	①Aspirin (100 mg/day) + famotidine (40 mg/day)	15	Aspirin (100 mg/day)	7	1. Number of participants with gastrointestinal lesions	None	Lansoprazole is superior to famotidine
			②Aspirin (100 mg/day) + lansoprazole (15 mg/day)				2. Side effect		
Akiko et al. (2010)	Japan	10	Aspirin (100 mg/day) + geranylgeranylacetone (150 mg/day)	10	Aspirin (100 mg/day)	7	1. Number of participants with gastrointestinal lesions	None	No significant difference between the two groups
							2. Side effect		
M. T. Donnelly et al. (2000)	UK	16	Aspirin (300 mg/day) + misoprostol (100 μg/day)	16	Aspirin (300 mg/day)	28	1. Number of participants with gastrointestinal lesions	3 in intervention group; 6 in control group	Misoprostol is superior to placebo
							2. Side effect		
Yeoh et al. (1995)	Singapore	18	Aspirin (600 mg/day) + chili	18	Aspirin (600 mg/day)	1	1. Number of participants with gastrointestinal lesions	None	Chili is superior to placebo
							2. Side effect		
Scheiman et al. (1994)	USA	20	Aspirin (2,600 mg/day) + omeprazole (40 mg/day)	20	Aspirin (2,600 mg/day)	14	1. Number of participants with gastrointestinal lesions	16 in intervention group; 7 in control group	Omeprazole is superior to placebo
							2. Side effect		
Hudson et al. (1993)	UK	21	Aspirin (1,800 mg/day) + ranitidine bismuth citrate (800 mg/day)	22	Aspirin (1,800 mg/day)	5	1. Number of participants with gastrointestinal lesions	None	Ranitidine bismuth citrate is superior to placebo
							2. Side effect		
Bergmann et al. (1992)	France	12	Aspirin (1,000 mg/day) + lansoprazole (30 mg/day)	12	Aspirin (1,000 mg/day)	7	1. Number of participants with gastrointestinal lesions	None	Lansoprazole is superior to placebo
							2. Side effect		

Table 1 summarizes the 10 trials published between 1992 and 2011. Most trials (8 of 10) were two-group studies, and only two trails had three or more groups. Overall, 503 patients were randomly assigned to one or two of the 10 gastrointestinal protecting medications or the placebo and included in the NMA. These RCTs compared 10 treatments, including omeprazole plus rebamipide (Kazuhiro et al., 2011), famotidine (Masafumi et al., 2011; Sugimoto et al., 2012), lansoprazole (Bergmann et al., 1992; Masafumi et al., 2011; Sugimoto et al., 2012), rabeprazole (Sugimoto et al., 2012), phosphatidylcholine complex (Byron et al., 2011), geranylgeranylacetone (Akiko et al., 2010), misoprostol (M. T. Donnelly et al., 2000), chili (K.G. Yeoh et al., 1995), omeprazole (James. M. Scheiman et al., 1994; Kazuhiro et al., 2011), and ranitidine bismuth citrate (N. Hudson et al., 1993). The mean study sample size was 50 participants, ranging from 20 to 181 participants. The treatment duration was less than 14 days in the vast majority of the subjects (80%).

### Quality of Trials

**FIGURE 3:**
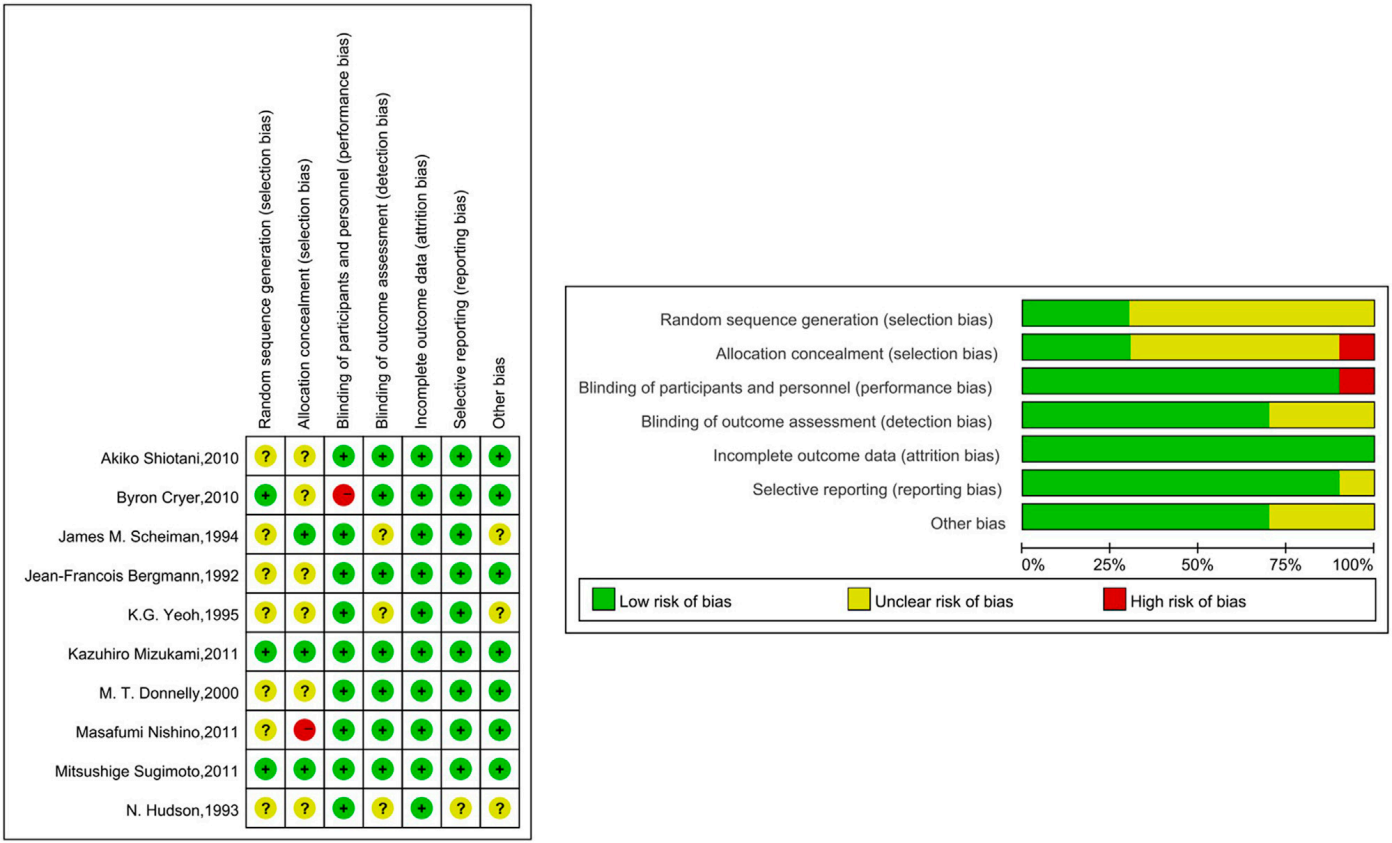
Risk of bias graph.

The quality of the studies included in this NMA is shown in Figure 3. In terms of random sequence generation and allocation concealment, 30% (3/10) were considered to be low risk. 90% (9/10) were rated as “low risk of bias” in terms of blinding of participants and personnel, and 70% (7/10) of the studies have the confirmation that the outcome indicators were not easily affected by subjective factors. All of the studies reported complete outcome data. 90% (9/10) of the studies were rated as “low risk of bias” in terms of selective reporting and 70% (7/10) of the studies were rated as “low risk of bias” in terms of other bias.

### Results of Meta-Analyses

**FIGURE 4:**
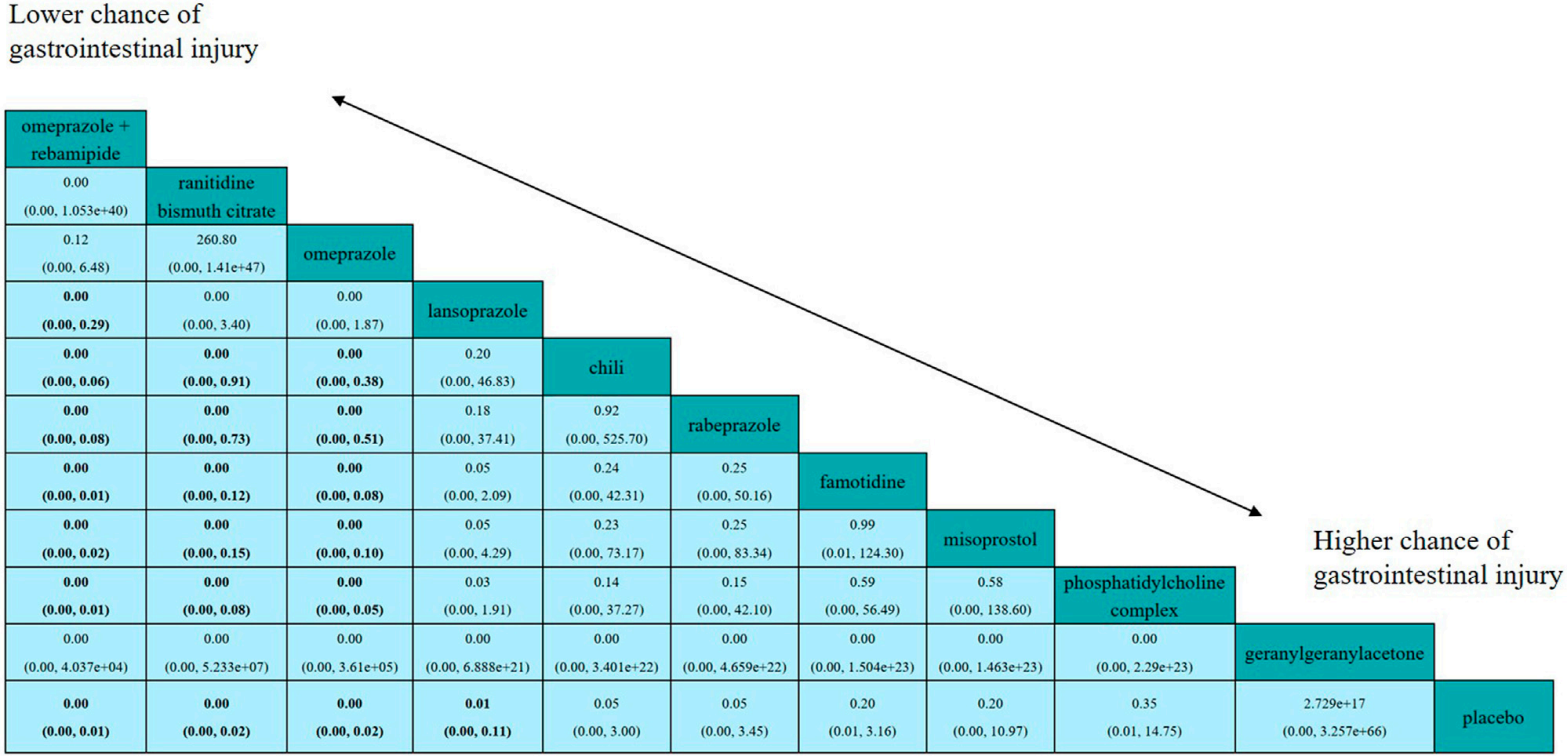
Efficacy of all interventions according to network meta-analysis. Drugs are reported in order according to efficacy ranking. Treatment at the top left corner ranks first, while the one at the bottom right corner ranks last. Comparisons between treatments should be read from left to right and the estimate is in the cell common between the column-defining treatment and the row-defining treatment. ORs (95% CrI) lower than one favor the column-defining treatment. Results are rounded to two decimal points. Significant results are in bold.

**FIGURE 5:**
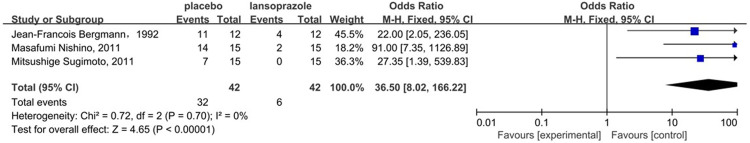
Direct meta-analysis.

Figure 4 shows the results of the head-to-head comparisons for the gastrointestinal injury. The treatments are reported according to their gastrointestinal injury ranking. Treatment at the top left corner ranks first, while the one at the bottom right corner ranks last. We estimated SUCRA values to rank all interventions for gastrointestinal injury. Omeprazole plus rebamipide had the highest number of significant differences compared with the other medications that are used clinically to prevent aspirin-induced gastrointestinal injury. Omeprazole plus rebamipide was associated with a significant reduction in the risk of gastrointestinal injury in pairwise comparisons with lansoprazole (OR: 0.00, 95% CI: 0.00–0.29), chili (OR: 0.00, 95% CI: 0.00–0.06), rabeprazole (OR: 0.00, 95% CI: 0.00–0.08), famotidine (OR: 0.00, 95% CI: 0.00–0.01), misoprostol (OR: 0.00, 95% CI: 0.00–0.02), phosphatidylcholine complex (OR: 0.00, 95% CI: 0.00–0.01), and placebo (OR: 0.00, 95% CI: 0.00–0.01). Ranitidine bismuth citrate was associated with a significant reduction in the risk of gastrointestinal injury in pairwise comparisons with chili (OR: 0.00, 95% CI: 0.00–0.91), rabeprazole (OR: 0.00, 95% CI: 0.00–0.73), famotidine (OR: 0.00, 95% CI: 0.00–0.12), misoprostol (OR: 0.00, 95% CI: 0.00–0.15), phosphatidylcholine complex (OR: 0.00, 95% CI: 0.00–0.08), and placebo (OR: 0.00, 95% CI: 0.00–0.02). Omeprazole was associated with a significant reduction in the risk of gastrointestinal injury in pairwise comparisons with chili (OR: 0.00, 95% CI: 0.00–0.38), rabeprazole (OR: 0.00, 95% CI: 0.00–0.51), famotidine (OR: 0.00, 95% CI: 0.00–0.08), misoprostol (OR: 0.00, 95% CI: 0.00–0.10), phosphatidylcholine complex (OR: 0.00, 95% CI: 0.00–0.05) and placebo (OR: 0.00, 95% CI: 0.00–0.02). Lansoprazole was associated with a significant reduction in the risk of gastrointestinal injury in pairwise comparisons with placebo (OR: 0.01, 95% CI: 0.00–0.11). In the direct meta-analysis, lansoprazole (OR, 36.50; 95% confidence interval [CI], 8.02–166.22) was also associated with the reduction in the risk of gastrointestinal injury compared to placebo (Figure 5).

**FIGURE 6:**
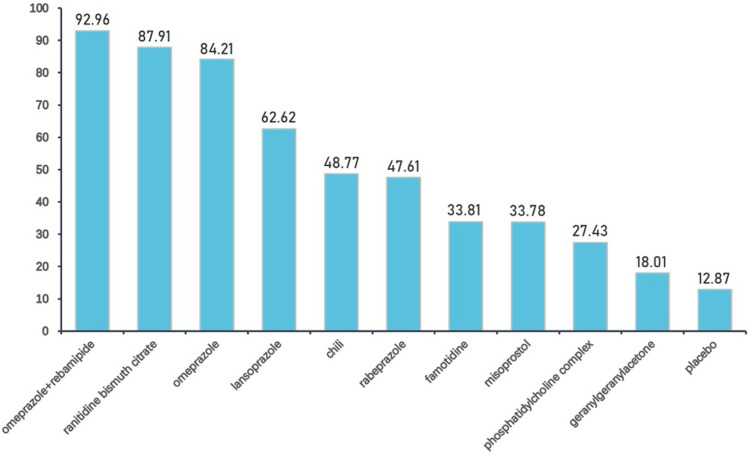
Interventions ordered by probability to be the best treatment. Drugs are ordered by their probability to be the best treatment in terms of preventing gastrointestinal injury. The cumulative percentages after normalization (0–100) are shown in the figure, with data from SUCRAs.

We further ranked all treatments according to SUCRA; Figure 6 presented all treatments ordered by their probability to be the best treatment in terms of gastrointestinal injury. It was suggested that omeprazole plus rebamipide, ranitidine bismuth citrate, and omeprazole outperformed other treatments, whereas geranylgeranylacetone and placebo were among the least treatments.

The inconsistency in the network for the nine interventions was summarized under consistency and inconsistency assumptions (Supplementary Tables); the difference of DIC between two models was no more than three, which means the results are generally considered consistent. In the heterogeneity analysis, global I-squared did not identified any heterogeneity across the studies (Supplementary Tables; global I ^2^ =  16.21).

## Discussion

Aspirin-induced gastrointestinal damage limits its regular use. PPI, H2RAs, misoprostol, alendronate, and some other gastrointestinal protecting medications are widely used in clinical practice for preventing the injury; however, there are no guideline recommendations for the injury. Therefore, identifying the treatment with the best safety and efficacy is important. To the best of our knowledge, there is currently no comparison of multiple interventions for treating aspirin-induced gastrointestinal injury; this is the first systematic review and network meta-analysis investigating different treatments for aspirin-induced gastrointestinal injury. Our study suggested that the combination of omeprazole and rebamipide was potentially the preferred intervention.

The mechanism of aspirin-induced gastrointestinal injury is usually considered to be divided into two parts: direct injury and indirect injury. The indirect mechanism of damage to the gastrointestinal mucosa by aspirin is the nonreversible inactivation of cyclooxygenase (COX), which inhibits the conversion of arachidonic acid to prostaglandin H2 (PGH2) in platelets (Hart and Bailey, 2002), subsequently affecting the production of prostaglandins PGE2 and PGI2. The decreased secretion of PGE2 decreases gastric mucus and HCO3-secretion and reduces gastrointestinal blood flow (Takeuchi, et al., 2015). The decreased secretion of PGI2 also decreases gastrointestinal lower blood flow (Harada et al., 1999). The limited energy caused by the lower blood flow reserved in the gastrointestinal mucosa makes it very sensitive to ischemia and hypoxia. It might cause damage to epithelial cells of the gastrointestinal mucosa, reduce mucosal barrier function, increase permeability and the susceptibility to endotoxin and bacterial attack. Another indirect mechanism of damage is that aspirin could affect intestinal flora, in particular, Gram-negative bacteria. It stimulates the immune system characterized by Toll-like receptor 4 and finally induces inflammation (Otani et al., 2017). The main ways in which aspirin causes direct gastrointestinal damage include *1*) disrupting the hydrophobicity of mucus HCO_3_^−^ protective layer of the gastric mucosa (Goddard and Hills, 1987); *2*) damaging the gastrointestinal mucosal epithelial cells, including inducing oxidative stress damage to the gastrointestinal mucosal epithelial cells, elevating malondialdehyde concentration and inducing apoptosis in the gastrointestinal mucosal epithelial cells (Hernandez et al., 2016); and *3*) disrupting the permeability of the gastrointestinal tract and causing inflammation (Lai et al., 2015). In summary, aspirin-induced gastrointestinal damage may be caused by a combination of inhibition of mucus secretion, inhibition of mucosal blood flow, oxidative stress damage, and inflammation, which is observed in our previous preclinical research (Zhu, et al., 2018). In such cases, inhibition of gastric acid production alone may not fundamentally inhibit aspirin-induced gastrointestinal injury. The treatment should be considered to elevate mucosal blood flow, reduce inflammatory infiltration, and promote mucus production at meantime. In animal experiments, rebamipide inhibited the infiltration of inflammatory cells in the gastric mucosa and increased the amount of gastric mucus, gastric mucosal blood flow, and gastric mucosal prostaglandin content. However, rebamipide showed no inhibitory effect on stimulating gastric acid secretion (Arakawa et al., 2005). Therefore, the combination of rebamipide with PPIs whose main effect is to inhibit gastric acid secretion can mechanistically inhibit aspirin-induced gastrointestinal injury.

H2RA and PPI are also used in clinical practice against aspirin-induced gastrointestinal injury. The results suggest that ranitidine bismuth citrate, omeprazole, and lansoprazole showed greater effectiveness than placebo. Ranitidine bismuth citrate is a combination drug of H2RA with bismuth citrate. The gastroprotective effects of ranitidine bismuth citrate include inhibiting gastric acid secretion (Tanaka, et al., 1996), inhibition of pepsin activity (Tay, et al., 1990), binding to ulcers (Koo et al., 1982), stimulation of prostaglandin synthesis and bicarbonate secretion (Konturek et al., 1987; Mertz-Nielsen et al., 1991), antibacterial activity (Manhart, 1990), and binding to mucus (Lambert, 1991). PPIs could inhibit gastric acid secretion and pepsin activity (El et al., 2018). Although not as good as the combination of rebamipide and PPIs, the use of PPI and ranitidine bismuth citrate alone can also be effective clinically.

In addition to the efficacy, safety is also an important issue in the treatment of aspirin-induced gastrointestinal injury. Clinicians have a responsibility to ensure that subjects were informed about the potential adverse drug reactions. The results showed that the combination of aspirin and protective treatments for gastrointestinal injury showed less side effects. Adverse events just happened in two treatments which are diarrhoea, dyspepsia, headache, aphthous ulcer, tinnitus, nausea, heartburn, abdominal pain, skin rash, and facial puffiness.

Compared with previous conventional pairwise meta-analyses, several merits of this study need to be highlighted. First, this study used network meta-analysis to explore the relative benefits of different treatments for aspirin-induced gastrointestinal injury and inform related clinical guidelines. The Bayesian model used in our study is currently the most applicable approach for multiple-intervention network meta-analysis, which could address the absence of direct comparison and reveal a favorable intervention by ranking analysis. Second, our literature search was as comprehensive as possible, which not only retrieved electronic databases commonly used in Chinese and English but also retrieved references from the reference lists of included RCTs and previous systematic reviews. However, this study also has some limitations, such as the uneven chronological distribution of the included literature, while the doses of aspirin used in some 1990s literature are too large compared to the current treatment regimen.

As a result, this comprehensive search strategy for the retrieval of the maximum number of available published trials and a predesigned inclusion criterion ensured the lowest possible degree of heterogeneity. There are several implications and considerations related to these findings. First, this study was conducted in healthy subjects to provide clinical indications for subsequent long-term users of aspirin. Second, this paper includes a variety of drugs that may play a role in prevention and treatment of aspirin-induced gastrointestinal injury, including novel drugs, to inform future clinical studies and guideline development.

## Conclusion

This is the first systematic review and network meta-analysis of different treatments for aspirin-induced gastrointestinal injury. The findings from this network meta-analysis represent the most comprehensive analysis of the available evidence. Our findings suggested that omeprazole plus rebamipide might be considered the best option to treat aspirin-induced gastrointestinal injury. These results should be taken into account in future guidelines and the selection of interventions for aspirin-induced gastrointestinal injury. Besides, more multicenter, high quality, large sample size randomized controlled trials will confirm the advantages of different medicine in the treatment of aspirin-induced gastrointestinal injury in the future.

## Data Availability

The original contributions presented in the study are included in the article/Supplementary Material, further inquiries can be directed to the corresponding authors.

## Abbreviations

AE, adverse event; COX, cyclooxygenase; GI, gastrointestinal; H2RA, histamine 2 receptor antagonist; NMA, network meta-analysis; PGE2, prostaglandins E2; PGH2, prostaglandin H2; PGI2, prostaglandins I2; PPI, proton pump inhibitor; PRISMA, Preferred Reporting Items for Systematic Reviews and Meta-Analysis; RCT, randomised controlled trials; SUCRA, Surface under the Cumulative Ranking curve.
