# Supplementary material for: Inhibition of Aspirin-Induced Gastrointestinal Injury: Systematic Review and Network Meta-Analysis
Source: Front Pharmacol. 2021 Aug 12;12:730681. doi: 10.3389/fphar.2021.730681 (PMC8406693; doi:10.3389/fphar.2021.730681)
Supplement: Supplementary file 1 [file Table1.docx]

Supplementary Table: A comparison of consistency and inconsistency model

a)

| Consistency model | | | Inconsistency model | | |
| --- | --- | --- | --- | --- | --- |
| DIC | Ratio | I^2 | DIC | Ratio | I^2 |
| 42.20204 | 0.854 | 0% | 43.30067 | 0.8519 | 0% |

b)

| Global I-squared: | |
| --- | --- |
| i2.pair | i2.cons |
| 17.47235 | 16.20449 |
